# Malaria and associated factors among pregnant women in Sekyere East District Ghana: a cross-sectional survey

**DOI:** 10.11604/pamj.2025.50.72.43079

**Published:** 2025-03-13

**Authors:** Victoria Bam, Bernard Gyamfi Anane

**Affiliations:** 1 School of Nursing and Midwifery, Kwame Nkrumah University of Science and Technology, Kumasi, Ghana

**Keywords:** Malaria, pregnancy, determinants, effects, Ghana

## Abstract

**Introduction:**

malaria in pregnancy (MiP) has deleterious effects not only on pregnant women but also on fetuses. This study therefore sought to assess the prevalence and associated factors of MiP in the Sekyere East District.

**Methods:**

a hospital-based cross-sectional study was conducted among pregnant women attending ANC at the Effiduase Government Hospital from June to August 2023. Malaria parasitaemia was detected using Giemsa-stained blood smear microscopy. Logistic regression models were used to test the association between the outcome and explanatory variables. Explanatory variables that were significant in univariate analysis at p values < 0.05 were included in the multivariate model. Variables with p values < 0.05 at the 95% confidence interval from the multivariable model were considered to be significantly associated with the outcome variable.

**Results:**

the prevalence of MiP was 29.8% (79/265). Being a primigravida (AOR=4.72, 95% CI: [1.89-11.79]; p=0.001), being in the third trimester of gestation (AOR=7.93, 95% CI: [2.18-28.83]; p=0.002) and the intake of 1 dose of IPTp-SP (AOR=6.37; 95% CI: [0.61-66.63]; p=0.029) were independently associated with increased odds of MiP.

**Conclusion:**

the prevalence of MiP is relatively higher in the Sekyere East District compared to other parts of Ghana. Gravidity, gestational age and doses of IPTp-SP taken are associated risk factors for MiP. Targeted health promotion programs are needed, especially for primigravidae and pregnant women in the latter stages of gestation, to reduce MiP in the Sekyere East District.

## Introduction

Malaria is a global public health burden, affecting approximately 2 billion people of all ages between 2000 and 2021 [1]. Globally, approximately 1.2 billion people are at risk of malaria, and an estimated 247 million cases of malaria and 619000 malaria-related deaths occur annually [2]. This is of enormous concern in sub-Saharan Africa [SSA]. This is because SSA contributes 95% of all malaria cases and 96% of all malaria-related mortalities [1]. Ghana has not been spared malaria. The country´s population of over 32 million people is at risk of malaria [3], and malaria is endemic in all parts of Ghana [4]. Ghana is also among the 15 malaria burden countries in the world, accounting for 2.1% and 1.9% of all global malaria morbidities and mortalities, respectively [2]. Pregnant women are among the most vulnerable groups to malaria infections [2]. Each year, 121.7 million pregnancies, representing 71% of all pregnancies in malaria-endemic areas and 49.2% of all pregnancies worldwide, are at risk of malaria infection [5,6]. Out of these pregnancies, 1.4 million (1.1%]) result in stillbirths, 33.5 million [27.5%] result in induced abortion, and 16.1 million [13.7%] result in miscarriage [6]. The WHO Africa Region accounts for approximately two-fifths (38%) of this total burden [2].

In Ghana, MiP accounts for 17.6% of outpatient department attendance, 13.7% of hospital admissions among pregnant women, 3.4% of maternal deaths, and 5% of all fetal deaths [7]. It exposes pregnant women and developing fetuses to health consequences such as maternal and fetal anaemia, stillbirth, preterm birth, and fetal growth restriction [2-4,8,9]. Given the dire health consequences associated with MiP, WHO recommended that all pregnant women adhere to strict practices of anti-malaria prevention measures [10]. These measures include the distribution and use of insecticide-treated bed nets (ITNs), intermittent preventive treatment (IPT) with sulfadoxine-pyrimethamine (SP) via directly observed therapy (DOT), and prompt diagnosis and treatment of infection [2,11,12]. The implementation of IPTp-SP and ITNs has been shown to reduce maternal malaria parasitaemia by 38%, malaria-related anaemia by 47%, low birth weight by 23%, miscarriages/stillbirths by 33%, and placental parasitaemia by 23% [4]. It has also been reported that if IPTp3 coverage is optimized to 90% of all pregnant women, 206500 low birth weights would be averted [2]. However, IPTs-SP are usually provided in the second trimester of pregnancy because of the contraindication of sulfadoxine-pyrimethamine due to its potential teratogenicity when used in the first trimester [13,14]. Moreover, ITNs are usually distributed to pregnant women at their first antenatal care (ANC) visit at approximately 4-5 months of pregnancy [15,16]. Studies have also reported low uptake of IPTp-SP and ITNs [10,14] and poor utilization of IPTp-SP via DOT [17]. Therefore, pregnant women may be insufficiently protected or not protected against malaria. There is a need to intensify efforts to control MiP and this would require the availability of accurate and reliable data on the MiP and associated across all parts of the world [4].

Undeniably, MiP prevalence and associated risk factors have received scholarly attention in sub-Saharan Africa. Studies have reported a MiP prevalence rate of 14.1 in northern Ghana [18], 16.4% in the Coastal Savannah [7], and 10.8% in western Ethiopia [16]. Factors such as gravidity [4,19,20], maternal age [4,16,21], gestational age [16,19,22], and level of utilization of ITNs [2,16,20,23,24] and IPTp-SP [19,24,25] have been revealed as risk factors for MiP. However, studies on the topic focusing directly on pregnant women in Sekyere East District are scarce, and MiP and associated factors in the district are poorly understood. Given that the MiP prevalence and associated factors vary with setting, it is also uncertain whether the determinants of MiP reported in other studies/settings influence MiP in the Sekyere East District. To address this research gap, the study assessed the MiP and associated factors in the Sekyere East District, Ghana.

## Methods

**Study design and setting:** this is a hospital-based cross-sectional study conducted at the antenatal clinic of the Effiduase Government Hospital from June to August 2023. The cross-sectional study was implemented using a quantitative research approach. Effiduase Government Hospital is a public health facility centrally located at Effiduase, the administrative capital of the Sekyere East Municipal Assembly in the Ashanti Region of Ghana. It is a 60-bed capacity facility and provides a 24-hour health service to approximately 60,000 indigenes of Effiduase and its neighboring communities [26]. It also serves as the major referral centre for emergency cases from various clinics and health centres within the district. The antenatal clinic where the study was conducted offers antenatal services from 8 am-3 pm on Mondays-Fridays. The unit sees approximately 50 antenatal clients each day. Care rendered at the ANC unit includes history taking, physical and obstetric assessment, weight, height, and blood pressure monitoring, HIV/AIDS screening and counselling, malaria, and anemia tests, etc. The geographical coordinates of Effiduase are latitudes 6°45" and 6°55" north and longitudes 1°15" and 1°25" west [26]. The climate of Effiduase is consistent with the general conditions seen in Ghana's central belt. With a monthly mean temperature of 26°C, it is typically warm. There are two distinct seasons [27]. The rainy season begins in April and ends in October or early November. The dry season starts from November to March. The rainfall pattern is bimodal, with a minor rainy season from September to early November and a major one from April to July [27]. The warm, reasonably humid weather is conducive to the life cycle of the female Anopheles mosquito, the main vector for malaria infection, in the district [26].

**Population and selection criteria:** the source population for our study was all consenting pregnant women receiving ANC care at Effiduase Government Hospital from June to August 2023, with records of malaria tests and the effect of MiP. Pregnant women with extremely debilitating conditions were excluded from this study. Pregnant women who registered with the Effiduase Government Hospital but were not residents of the Sekyere East District were excluded from the study to ensure that reported prevalence rates of malaria represent an accurate reflection of the true status of malaria prevalence among participants from the study area only.

**Sample size determination:** the sample size was determined using the Cochran formula [28]:


$$\text{N}=\frac{Z^{2}pq}{d^{2}}$$

at 19.8% prevalence of MiP in Ashanti Region [29], 95% confidence interval (CI), and 5% margin of error. To compensate for the noncompleteness rate and to strengthen statistical power, 15% of the calculated sample size was added, which gave a total sample size of 280.

**Sampling technique:** the pregnant women receiving antenatal care at Effiduase Government Hospital were approached directly and solicited during and after the ANC services to participate in the study. The pregnant women were adequately informed about the purpose and scope of the study. Using the nonprobability convenient sampling technique, willing and consenting pregnant women, who satisfied the selection criteria, were enrolled in the study.

**Variable measurement:** the dependent variable in this study was the presence of malaria parasitaemia assessed with light microscopy of blood films (thick and thin). The presence of any type of Plasmodium species from the examination was considered malaria infection. The independent variables in this study were the participants´ demographic characteristics *(age, marital status, religion, employment status etc.)*, obstetric and reproductive history *(gestational age, gravidity, parity)*, malaria preventive measures *(owning bet nets, use of SP)* and effect of malaria infection (documented symptoms and complications of MiP).

**Data source and data collection procedure:** the study employed two main approaches for the data collection. Primary data such as maternal age, educational level, and use of ITNs were obtained by administering pretested structured questionnaires, and secondary data such as the number of ANC visits, current gestational age, gravidity and parity, records on administration of IPTp-SP and malaria test results were extracted from the participants´ ANC Booklets and Medical Records. The medical, laboratory, and obstetric records of the malaria-infected pregnant women were reviewed. Records of complications of MiP, such as stillbirth, threatened abortion, premature delivery, severe anaemia (HB < 7g/dL), etc. and symptoms of MiP such as vomiting, headache, and generalized body weakness, etc., were considered the effects of MiP [9,30]. Two trained research assistants, under the supervision of the principal investigator, did the data collection.

### Laboratory investigation

**Blood films for malaria parasitaemia:** thick and thin smears were prepared from the peripheral blood of ANC clients. The films (thin film fixed in methanol) were Giemsa stained (10%) and examined under a light microscope at 100x magnification. The thick film was used for parasite quantification and identification, while the thin film was used for species differentiation. Two experienced medical laboratory scientists read all the slides independently. A film was said to be positive when both medical laboratory scientists recorded a positive result. A film was considered negative only if no parasite was seen on a thick film after observing at least 100 high-power fields (HPF). In instances where a 10% inconsistency occurred, a third reader was required to confirm the diagnosis.

**Haemoglobin estimation:** Haemoglobin (HB) concentration was estimated using a HemoCue analyzer (HemoCue Hb 201+). 20µL of capillary or venous whole blood was pipetted onto the HemoCue analyzer for HB estimation. Using the WHO criteria, HB concentration of <11g/dL was considered anaemia in pregnancy (AiP). AiP was further classified as mild, moderate, and severe at HB concentrations of 10-10.9g/dL, 7-9.9g/dL, and < 7g/dL respectively [31].

**Data processing and analysis:** data obtained were carefully checked for correctness and inconsistencies were resolved before analysis. Data were entered and cleaned in MS Excel and later imported into SPSS version 25 for data analysis. Descriptive statistics for categorical variables such as level of education, etc. were presented in frequencies and percentages whereas continuous variables such as monthly income, and HB concentration were presented as mean ± SD or median (interquartile range), where appropriate. To compare the haemoglobin concentration between participants with malaria infection and those without malaria infection, an independent two-sample t-test was performed. The assumption of normality and homogeneity of variance were satisfied. All tests were two-sided and a p-value < 0.05 was considered statistically significant. To determine the factors associated with MIP, we first performed a univariable logistic regression analysis. Variables that were statistically significant in univariable analysis at a 95% confidence interval and p< 0.05 were incorporated into the multivariable model to control the possible effects of confounders. Multicollinearity was examined using a maximum variance inflation factor (VIF) of 4. Parity was highly correlated with gravidity and was thus removed from the final multivariate model. The model goodness of fit was measured using the Hosmer-Lemeshow test (0.712). Independent variables that were significant in the final multivariate model on the best of adjusted odds ratio (AOR), with 95% CI and p-value < 0.05, were considered the independent factors associated with MiP.

**Ethical approval and consent to participate:** ethical clearance was obtained from the Committee on Human Research, Publication, and Ethics (CHRPE), KNUST with a reference number CHRPE/AP/504/23. Administrative approval was obtained from the Management of Effiduase Government Hospital. Written consent was sought from each participant. The data collection procedure was anonymous. The participants were informed of the right to opt out of the study without incurring any medical or financial repercussions. Data were handled with extreme confidentiality.

## Results

**Sociodemographic and obstetric characteristics of the participants:** a total of 265 pregnant women participated in the study. The mean age ± SD (range) of the participants was 29.23±6.14 years, ranging from 19 years to 40 years. The majority (46.8%) of the participants were within the age range of 25-34 years, professed the Christian faith (85%), were married (58.1%), and lived in communities outside Effiduase (55.5%). The mean monthly income ± SD (range) of the participants was GH₵ 767.93±897 (GH₵0-5000) and nearly a third of the participants (31.3%) earned between GH₵500-GH₵999 monthly. Nearly half of the participants (49.4%) were in their third trimester of gestation, while 38.5% were multigravidas. The majority of the participants (88%) attended ANC appointments as scheduled, two-thirds of the participants (65.3%) had attended ANC at least 4 times, and more than half of the participants (55.8%) had their first ANC within their 13^th^-28^th^ week of gestation. The socio-demographic and obstetric characteristics of the participants are presented in Table 1.

**Table 1:** socio-demographic and obstetric characteristics of pregnant women in the Sekyere East District, Ghana

Variables		Frequency (n=265)	Percentage (%)
**Age (Years)**	19-24	87	32.8
**Mean age ±SD (Range) 29.23±6.14 (19-42)**			
	25-34	124	46.8
	≥35	54	20.4
**Religion**	Christian	225	85.0
	Muslim	40	15.0
**Marital Status**	Single	34	12.8
	Married	154	58.1
	Cohabiting	4	1.6
	Other (divorced, widowed)	73	27.5
**Residence**	Effiduase	118	44.5
	Outside Effiduase	147	55.5
**Employment Status**	Employed	70	26.4
	Self-employed	140	52.8
	Unemployed	55	20.8
**Highest educational status**	No formal education	16	5.7
	Below SHS	114	43.0
	Senior SHS	135	52.3
**Monthly income (GH₵)**	None	55	20.8
**Mean income ±SD (Range), 767.93±897.40 (0-5000)**			
	<GH₵500	76	28.7
	GH₵500-999	83	31.3
	≥GH₵1000	52	19.2
**Gestational age**	First trimester	44	15.6
	Second trimester	90	34.0
	Third trimester	131	49.4
**Gravidity**	Primigravidae (1)	84	31.7
	Secundigravidae (2)	79	29.8
	Multigravidae (3 or more)	102	38.5
**Number of ANC scheduled appointments attended**	< 4 times	92	34.7
	≥4 times	173	65.3

SHS-Senior High School, GH₵-Ghanaian Cedi, SD: Standard deviation

**Malaria preventive practices of participants:** Table 2 below summarizes the malaria preventive practices of the participants. The majority of the participants, 248 (93.6%), had received ANC counselling and health education on malaria preventive measures. The majority of the participants, 185 (70%), were on the IPTp-SP program. Of these (185), 40% had received one dose of IPTp-SP, while one-third (32%) had received 2 doses of IPTp-SP. However, 80 (30%) of the participants were not on the IPTp-SP program. Being in the first trimester of gestation (55%), experiencing undesired effects of IPTp-SP during previous pregnancy (34%), and being positive for G6PD (11%) were the reported reasons for non-utilization of the IPTp-SP program. Over two-thirds of the participants, 182 (68.7%), were ITN users. Of the ITN users (182), 77% use LLINs and 23% use ordinary bed nets. The majority of the participants did not use mosquito repellent (93.2%), mosquito coil (83%), or IRS (89%).

**Table 2:** malaria preventive measures employed by pregnant women in the Sekyere East District, Ghana

Variables	Frequencies (n=265)	Percentage (%)
**ANC counselling and health education on malaria preventive practices**		
Yes	248	93.6
No	17	6.4
**Form of education on MIP received at the ANC (n=248)**		
Individual	21	8.5
Group	137	55.2
Both	90	36.3
**IPTp-SP use**		
Yes	185	70.0
No	80	30.0
**Doses of IPTp-SP received (n=185)**		
1	74	40.0
2	60	32.0
3	44	24.0
>3	7	4.0
**Reason for no intake of IPTp-SP (n=80)**		
Positive for G6PD	9	11.0
Currently at 1st trimester	44	55.0
Undesired effects of IPTp-SP	27	34.0
**Reception of ITN from ANC**		
Yes	158	59.6
No	107	40.4
**Utilization of ITN**		
Yes	182	68.7
No	83	31.3
**Frequency of ITN use (n=182)**		
Always (5-7 times per week)	137	75.3
Sometimes (3-4times per week)	41	22.5
Rarely (<3 times per week)	4	2.2
**Type of ITN Used (n=182)**		
Ordinary net	42	23.0
LLINs	140	77.0
**Repellent Use**		
Yes	18	6.8
No	247	93.2
**Mosquito Coil**		
Yes	45	17.0
No	220	83.0
**IRS**		
Yes	29	11.0
No	236	89.0

IRS-Indoor Residual Spraying, LLINs-Long Lasting Insecticides Nets, ITN-Insecticides Treated Nets

**Figure 1:**
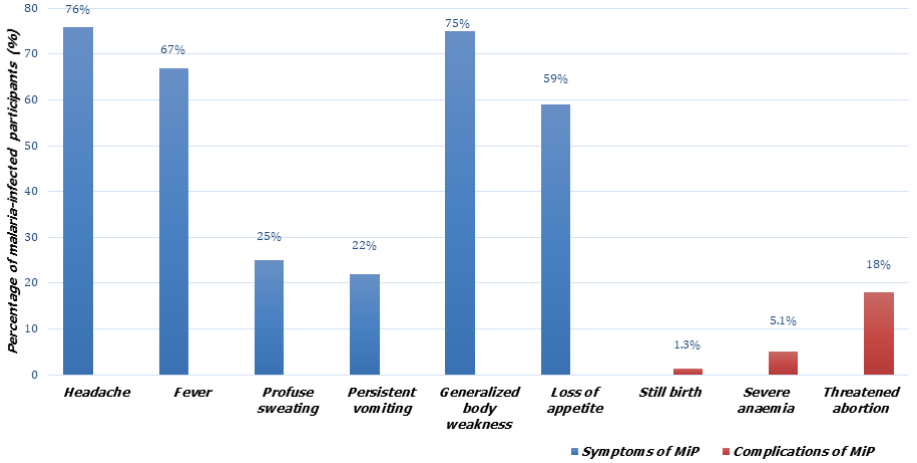
effects of malaria among pregnant women in the Sekyere East District, Ghana

**Prevalence and effects of MiP:** Table 3 and Figure 1 summarize the prevalence and effects of MiP. The prevalence rate of MiP was 79 (29.8%). The overall prevalence of anaemia was 40.4%, with 22.2%, 14.0%, and 4.2% of the participants presenting with mild, moderate, and severe anaemia respectively. One-third of the malaria-infected participants 26 (33%) had anaemia with 20.3%, 7.6%, and 5.1% presenting with mild, moderate, and severe anaemia respectively. With respect to the effects of MiP, the majority of the malaria-infected participants 74 (93.6%) experienced at least a symptom of MiP (Table 3) with 76%, 75%, and 67% presenting with headache, generalized body weakness, and fever respectively (Figure 1). A total of 19 (24.1%) malaria-infected participants experienced complications of MiP (Table 3) with 18.0%, 5.1%, and 1.3% experiencing threatened abortion, severe anaemia, and stillbirth respectively (Figure 1).

**Table 3:** medical and laboratory records of pregnant women in the Sekyere East District, Ghana

Variables			Frequencies (n=265)			Percentage (%)		
**Presence of malaria parasitaemia**		Yes	79			29.8		
		No	186			70.2		
**Was MiP symptomatic? (n=79)**		Yes	74			93.6		
		No	5			6.4		
**Did you experience any complications of MIP? (n=79)**		Yes	37			46.8		
		No	42			53.2		
**Hb Concentration (g/dL)**		≥11.0g/dL	158			59.6		
**Mean±SD (Range)= 10.48±2.19 (6.20-14.00)**								
		9-10.9g/dL	59			22.2		
		7-8.9g/dL	37			14.0		
		7g/dL)	11			4.2		
**Hb concentration of malaria infected participants(g/dL) (n=79)**		≥11.0g/dL	53			67.0		
		<11.0g/dL	26			33.0		
**Classification of anaemia in malaria infected participants (n=26)**		Mild	16			20.2		
		Moderate	6			7.6		
		Severe	4			5.1		
	**Hb concentration**		**t test for equality of means**			**95% Confidence Interval**		
**Malaria parasitaemia**	**n**	**Mean±SD**	**df**	**T**	**Sig level (2 tailed)**	**Lower limit**	**Upper limit**	
Yes	79	9.70±2.15	263	3.895	0.000	0.55	1.67	
No	186	10.81±2.13	-	-	-	-	-	
**Total**	265	10.48±2.19	-	-	-	-	-	

Hb-Haemoglobin, MiP-Malaria in Pregnancy

**Haemoglobin concentration in malaria and non-malaria infected participants:** the mean HB concentration of the participants was 10.48±2.19, ranging from 6.20g/dL to 14.00 g/dL. The mean ± SD HB concentration of participants with MIP and participants without MiP were 9.70±2.15 g/dL and 10.81±2.13 g/dL respectively. The mean HB difference between participants with MiP and participants without MiP was statistically significant (t=3.895, df=263, sig level=0.001). See details in Table 3 below.

**Factors associated with MiP:** the unadjusted and adjusted results are presented in Table 4. In the unadjusted/univariate analysis, MIP was more likely to occur in pregnant women in their 3^rd^ trimester of gestation (COR=3.43; 95% CI: [1.35-8.71]; p=0.038), those who were primigravidas (COR=3.86; 95% CI: [1.98-7.52]; p=0.001), those on the IPTp-SP program (COR=1.62; 95% CI: [0.18-14.38]; p=0.045), those who had received one dose of IPTp-SP (COR=4.57; 95% CI: [0.53-39.89]; p=0.003) and those within the age bracket of 19-24 years (COR=2.36; 95% CI: [1.09-5.10]; p=0.01). In the adjusted/multivariate analysis, being in 3rd trimester of gestation (AOR=7.93, 95% CI: [2.18-28.83]; p=0.002), primigravida (AOR=4.72, 95% CI: [1.89-11.79]; p=0.001) and having received only one dose of IPTp-SP (AOR=6.37; 95% CI: [0.61-66.63]; p=0.029) were independently associated with increased odds of MiP. Although univariate analysis showed that maternal age was inversely associated with MiP, it was unable to independently predict the risk of MiP after controlling for possible confounders (maternal age;19-24: AOR=1.78; 95% CI: [0.58-5.58], p=0.324). Similarly, being on the IPTp-SP program showed a significant association with MiP, but it was unable to independently predict the risk of MiP after adjusting for possible confounders (being on the IPTp-SP program: AOR=2.46; 95% CI: [0.22-27.98], p=0.469)

**Table 4:** univariate and multivariate regression analysis of factors associated with MiP in Sekyere East District, Ghana

Variable	Malaria Parasitaemia		Crude odds ratio (95% CI)	P value	Adjusted odds ratio (95% CI)	P value
	Yes	No				
**Age**				0.031		
19-24	35	52	2.36(1.09-5.10)	0.010*	1.78 (0.58-5.58)	0.324
25-34	32	92	1.22(0.57-2.60)	0.181	1.45(.57-3.70)	0.442
≥35	12	42	1		1	
**Gestational age**				0.026		
1st trimester	6	38	1		1	
2nd trimester	27	63	2.71(1.03-7.15)	0.962	2.17(0.69-6.79)	0.183
3rd trimester	46	85	3.43(1.35-8.71)	0.038*	7.93(2.18-28.83)	0.002*
**Gravidity**				0.001		
Primigravidae	38	46	3.86(1.98-7.52)	0.001*	4.72(1.89-11.79)	0.001*
Secundigravidae	23	56	1.92(0.95-3.87)	0.872	2.03(0.90-4.57)	0.089
Multigravidae	18	84	1		1	
**IPTp-SP Program**				0.045		
Yes	62	123	1.62(0.18-14.38)	0.045	2.46(0.22-27.98)	0.469
No	17	63	1		1	
**Doses of IPTp-SP intake**				0.033		
1	32	42	4.57(0.53-39.89)	0.003*	6.37(0.61-66.63)	0.029*
2	18	43	2.57(0.29-22.93)	0.971	1.83(0.18-18.61)	0.611
3	11	33	2.00(0.22-18.49)	0.445	1.56(0.150-16.43)	0.710
More than 3	1	6	1		1	

NB: * denote p<0.05 statistically significant, COR and AOR of 1 denote the reference category

## Discussion

**Prevalence of MiP:** the study assessed the prevalence of MiP among pregnant women seeking ANC care at the Effiduase Government Hospital. The prevalence of MiP was 29.8%. This figure is comparable to what was reported at Agona Hospital, Ghana (28.3%) [29], Ndirande Health Centre-Blantyre, Malawi (28.4%) [15], and Mali [32]. Effiduase Government Hospital, Agona Hospital, and Ndirande Health Centre are in peri-urban communities. These facilities are patronized by clients from neighboring rural communities where environmental factors suitable for vector breeding, such as poor sanitation are common, and proper malaria control measures, such as residual spraying, are rare. These reasons may explain the conformity of the reported prevalence of MiP.

Conversely, the prevalence of MiP in our study is higher than what was reported at Dodowa Government Hospital (11.1%) [33], Greater Accra Regional Hospital (5.5%) [34], southern Laos, Nigeria (5.9%) [8], Arba Minch-South Ethiopia (18.8%) [20] and Benin (20.8%) [14]. These findings suggest intra-, inter-regional, and international variations in the reported malaria prevalence rates and could be attributed to the effect of geographical location on malaria endemicity. In different geographical locations, there may be different malaria transmission levels and unique transmission patterns, which may result in different immune acquisition capacities of the indigenes. The discrepancy could also be attributed to differences in the inclusion criteria, seasonal variations, diagnostic tests, and levels of adherence to malaria preventive measures. For instance, our study was carried out within June-August. This season was characterized by adequate rainfall, humidity, and warmth, which are environmental conditions that enhance the breeding, growth, and survival of mosquitoes and transmission of malaria and thus could account for the relatively higher prevalence of MiP. Again, unlike studies that considered only asymptomatic MiP [20,34], MiP in the first trimester [14], or MiP at the first ANC visit [4], our study considered all malaria infections, regardless of the presence/absence of symptoms, gestational age, and antenatal visit. This may therefore explain the relatively higher observed prevalence of MiP in our study.

On the other hand, our prevalence of MiP of 29.8% was lower than what was reported in Navrongo, Ghana (41%) [35] and Ondo State, Nigeria (52.5%) [36]. In Ondo State, Olusi *et al*. [36] recruited HIV-positive pregnant women. The literature attests that HIV patients are susceptible to malaria infection due to reduced immunity resulting from the depletion of clusters of differentiation 4 (CD4) cells by the virus, which consequently reduces the production of malaria antibodies [37]. This may therefore explain the considerably higher prevalence of MiP in Ondo state [36]. Though microscopy remains the gold standard diagnostic test for malaria in Africa [38,39], its limited sensitivity compared to PCR is noteworthy [40,41]. The study used microscopy [a less sensitive tool] and recorded a prevalence rate of 29.8%, which surpassed the national prevalence rate of 17.2%. This finding warrants considerable concern.

**Factors associated with MiP:** the study assessed the associated risk factors for MiP. There was a significant association between MiP and gravidity. Primigravidae were at significantly higher risk of malaria infection than multigravidae. This finding corroborates the findings of studies carried out in Ghana [4,21,24], Ethiopia [20], and Nigeria [8]. Low odds of MiP in multigravidae could be linked to the development of pregnancy-related immunity against malaria infection from repetitive exposures during previous pregnancies, which the primigravidae were yet to acquire. Another reason could be that multigravidae had previous exposure to health services such as ANC teaching and counselling, gained a detailed understanding of the diseases, and are currently adopting proper malaria preventive measures. Gestational age was significantly associated with MiP. Pregnant women in the third trimester of gestation had higher chances of being infected with malaria than pregnant women in their first trimester of gestation. These findings align with the findings of a sub-Saharan African study [22] The increased odds of MiP with gestational age could be due to suppressed immunity associated with pregnancy. Immunosuppression associated with pregnancy may be pronounced in the third trimester, where there is usually rapid growth and development of the foetus.

However, our findings contradict the findings of studies conducted in the Hohoe Municipality-Ghana [19] and the Sherkole District-Ethiopia [16], which reported an inverse relationship between the odds of MiP and gestational age, highest in the first trimester and least in the third trimester. The reduced risk of MiP in the third trimester reported by Kweku *et al*. [19] and Gontie *et al*. [16] could be due to the impact of IPTp-SP and ITN use adopted during the 2nd trimester of gestation. Another possible reason for the reported significantly higher odds of MiP in the first trimester could be that the pregnant women had malaria infection that was subpatent but became microscopic during early pregnancy due to changes in women´s physiology and immunity.

Our findings showed that the dosage of IPTp-SP intake was independently associated with MiP. Pregnant women on the IPTp-SP program who had taken only one dose of IPTp-SP had significantly higher chances of developing malaria infection than pregnant women who had received at least three doses of IPTp-SP. These findings align with what was reported in studies conducted in Gabon [13] and Ghana [17]. These findings suggest that all pregnant women should genuinely adhere to at least three doses of IPTp-SP as recommended by the World Organization to gain IPTp-SP´s full protection against malaria infection [3].

**Effects of MiP on pregnant women:** our study assessed the effects of malaria infection on pregnant women. MiP was symptomatic and complicated. MiP was linked with symptoms such as headache, fatigue, and complications such as severe anaemia, threatened abortion, and stillbirth. These findings are consistent with the findings of studies conducted in North America [9] and sub-Saharan Africa [8,12,42], where MiP was associated with symptoms such as headache, chills, and complications such as severe anaemia and oligohydramnios. In contrast, Ahenkorah *et al*. [4] revealed that malaria-infected pregnant women experienced no symptoms or complications of the disease. The effects of MiP differ with areas of different transmission levels [35,43]. In areas of low transmission, pregnant women rarely develop immunity to malaria [35]. MiP is usually symptomatic and complicated [44]. However, in high transmission areas, pregnant women usually develop natural immunity to malaria infection due to frequent mosquito exposure, which consequently results in asymptomatic and uncomplicated malaria [44].

**Limitations:** a cross-sectional design was used. Due to this, the long-term effect of MiP on maternal and fetal health could not be studied. A longitudinal study should be carried out to elucidate the long-term effect of malaria infection on maternal and fetal health. Despite this limitation, the findings provide valuable information to support the countries´ malaria control efforts and that of countries with similar endemicity of malaria.

## Conclusion

The prevalence of MiP in the Sekyere East District is relatively higher compared to other findings cited in Ghana. Primigravidity, being in the third trimester of gestation and the intake of only one dose of IPTp-SP were independent associated risk factors for MiP. Targeted health promotion programs are needed, especially for primigravidae and pregnant women in the latter stages of gestation. Regular and intensified education on adherence to the recommended doses of IPTp-SP is needed to help minimize the risk of MiP.

### What is known about this topic

- Headache, vomiting, and generalized body pains are reported symptoms of MiP;
- Severe anaemia was a complication of MiP.

### What this study adds

- This study has shown that being a primigravida or being at the latter stages significantly increased the risk for MiP;
- The study revealed that utilization of ITN does not necessarily result in reduced risk of MiP as reported in other studies;
- Proper adherence to IPTp-SP (the uptake of at least three doses of IPTp-SP during pregnancy) is associated with reduced risk of MiP.
